# A Scientific Overview of Smartphone Applications and Electronic Devices for Weight Management in Adults

**DOI:** 10.3390/jpm9020031

**Published:** 2019-06-07

**Authors:** Sophie Laura Holzmann, Christina Holzapfel

**Affiliations:** Institute for Nutritional Medicine, Else Kroener-Fresenius-Centre for Nutritional Medicine, School of Medicine, Klinikum rechts der Isar, Technical University of Munich, 81675 Munich, Germany; sophie.holzmann@tum.de

**Keywords:** mobile applications, wearables, electronic devices, nutrition, physical activity, lifestyle, overweight, obesity, weight management

## Abstract

Worldwide, there are rising trends in overweight and obesity. Therefore, novel digital tools are discussed to improve health-related behaviors. The use of smartphone applications (apps) and wearables (e.g., activity trackers) for self-monitoring of diet and physical activity might have an impact on body weight. By now, the scientific evaluation of apps and wearables for weight management is limited. Although some intervention studies have already investigated the efficacy of aforementioned digital tools on weight management, there are no clear recommendations for its clinical and therapeutic use. Besides the lack in long-term randomized controlled trials, there are also concerns regarding the scientific quality of apps and wearables (e.g., no standards for development and evaluation). Therefore, the objective of present work is: (1) To address challenges and concerns regarding the current digital health market and (2) to provide a selective overview about intervention studies using apps and activity trackers for weight-related outcomes. Based on cited literature, the efficacy of apps and wearables on weight management is assessed. Finally, it is intended to derive potential recommendations for practical guidance.

## 1. Overweight and Obesity

**Figure 1:**
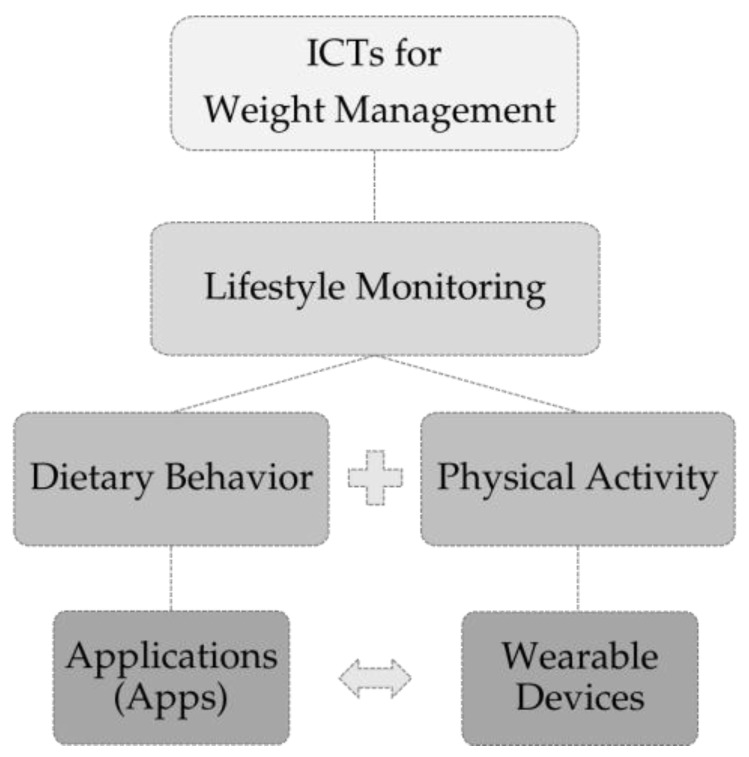
Novel information and communication technologies (ICTs) for lifestyle monitoring (diet; physical activity) and weight management. App = application on smartphones; wearable device = portable hardware/activity tracker.

The rising trends of overweight and obesity worldwide [1] are major public health concerns [2]. Since the 1970s, worldwide obesity increased almost threefold [3]. In 2016, nearly 2 billion adults were overweight, and around one-third of people were obese [3]. Besides an increase in energy-dense and nutrient-poor nutrition [1], there is also a decline in physical activity [4]. Both lifestyle factors are considered to be leading causes for the high prevalence of overweight and obesity [5]. Overweight and obesity increase the risk for co-morbidities (non-communicable diseases (NCDs)), such as cardiovascular diseases, diabetes, musculoskeletal disorders, and certain types of cancer [3]. Scientific research in the field of weight reduction often addresses individual responsibility, e.g., by improving diet and physical activity through the application of behavior change techniques. For instance, self-monitoring of diet and physical activity provides an effective behavior change technique for weight management. It was already shown that dietary self-monitoring is related to weight loss [6]. Figure 1 gives a general overview about the use of novel information and communication technologies (ICTs) for lifestyle monitoring and weight management. 

As traditional weight loss approaches may lack efficacy, including low compliance as well as high time and cost efforts, digitally delivered or supported lifestyle interventions are in the spotlight of current research [4,7]. 

## 2. Digital and Mobile (m) Health—Definition 

By now, there is no harmonized definition for terms like “digital health” or “mobile (m) Health” [8,9,10], thus leading to multiple operational definitions [9]. For instance, a systematic review on published definitions of electronic (e) Health showed that there are 51 unique definitions of eHealth [11]. According to the US Food and Drug Administration (FDA) digital health includes “categories such as mHealth, health information technology (IT), wearable devices, telehealth and telemedicine, and personalized medicine” [12]. Moreover, the Global Observatory for eHealth (GOe) by the World Health Organization (WHO) defines mHealth as “medical and public health practice supported by mobile devices, such as mobile phones, patient monitoring devices, personal digital assistants (PDAs), and other wireless devices” [10]. Throughout this article, the focus is on the following categories: “mobile health applications (apps)” and “wearables”, specifically “activity trackers”. 

## 3. Digital and Mobile (m) Health—Quality Assessment

Besides the absence of a standard definition of the aforementioned terms, there are further limitations and challenges that need to be addressed. For instance, there are no standards available for the development, evaluation, and certification of apps and digital devices. At the development stage, specialist expertise and information references are often absent or deficient [13,14,15,16], resulting in non-reliable and non-transparent contents [13,17,18,19,20]. However, this insufficiency also applies for data privacy statements and scientific evidence [14,16,20]. An evaluation of selected nutrition apps revealed that single nutrition values deviated by as much as 50% from nutrition values provided by the German Food Database [20]. 

In this context, it has to be emphasized that the following legal limitations do not refer to medical devices, for which distinct regulations apply (e.g., FDA). In Germany, for instance, medical devices are subject to the German Law of Medical Devices (Medizinproduktegesetz, MPG) and its corresponding regulations. Moreover, medical devices and medical apps need to be certified by the Federal Institute for Drugs and Medical Devices (Bundesinstitut für Arzneimittel und Medizinprodukte, BfArM). Therefore, the guidance on “Medical Apps”, enacted by the BfArM, is intended to support developers regarding the differentiation between medical devices and wellness applications (health and fitness apps) [21].

### 3.1. Development, Evaluation, and Certification 

In 2014, the European Commission published a “Green Paper on mHealth”, which addresses limitations of mHealth, e.g., data protection and security, transparency of information, scientific research, and considers its potential for the healthcare system and the mHealth market as well [22,23]. In addition, the European Commission is working on a “privacy code of conduct for mHealth apps”, including “practical guidance for app developers on data protection principles while developing mHealth apps”. Besides that, the WHO developed guidelines for the reporting of health interventions using mobile phones, the so-called “mHealth evidence reporting and assessment (mERA) checklist”, considering 16 core items, such as “data security” and “compliance with national guidelines” [24]. Moreover, the mHealth app guideline “Xcertia” was elaborated by a US healthcare collaborative and aims to ensure safe and effective health apps. The guideline covers the following app-related topics: operability, privacy, security, content, and usability [25]. Additionally, there are also validated questionnaires available for the quality assessment of health (mobile rating app scale, MARS) [26] and nutrition apps (app quality evaluation, AQEL) [27]. 

By now, several German scientific institutions and organizations have established quality seals, regulatory marks, orientation aids, guidelines, and recommendations for the attentive handling of apps [28,29,30,31,32,33,34,35,36,37]. For example, the information and evaluation platform “HealthOn” (www.healthon.de) evaluates and approves health apps by the “HealthOn code of honour”, a trust mark and quality seal [30,38]. The code relates to the reliability in-app health-related information and services and covers following criteria: (1) Authorship and medical accuracy, (2) actuality and relevance of the sources used, (3) advice “physician consultation”, (4) product and advertising freedom, (5) sources of finance, (6) data and consumer protection, and (7) voluntary self-control [30]. Although there are legal frameworks for the harmonization of European data protection, there are still insufficiencies regarding the implementation and modernization of the national data privacy law in the German health sector [39]. To get an overview about quality seals, regulatory marks, and orientation aids, Albrecht et al. investigated the metadata (store descriptions) of “medicine” and “health and fitness” apps available at Apple’s German App Store [29]. It was shown that only a few manufacturers referenced seals, primarily CE marks, demonstrating that there is no relevance of quality seals for app providers. Finally, the authors concluded that a “possibly legally obligatory, standardized reporting system should be implemented” [29].

### 3.2. Data Privacy, Data Reliability, and Expert Involvement

One of the most commonly addressed concerns are the acquisition, management, transmission, storage, protection, and privacy of collected data [28,39,40]. Although there are international legal frameworks aiming to ensure transparency and security of mHealth devices, app developers often neglect legal specifications, leading to persisting and reasonable concerns regarding privacy and data protection [40]. For instance, the recently enacted EU General Data Protection Regulation (GDPR) represents a “comprehensive legal framework” and “harmonizing bracket” regarding “consent, purpose binding and data transfer, rights of the data subject, technical and organizational measures, and procedural arrangements” [39]. However, at present there are no holistic, mandatory regulations for the development and release of apps. Literature indicates that, with regard to nutrition apps, imprint information in app stores and corresponding homepages are often missing [20]. Braz et al. evaluated 16 free available nutrition apps and verified the quality and reliability of nutritional information related to values of macronutrients, micronutrients, and energy [13]. It was concluded that investigated apps lack reliable sources of information and therefore are not recommended for nutritional guidance [13]. Moreover, data security statements and privacy policies of health apps are often deficient, non-transparent, incomprehensible, or even missing [41,42], especially with regard to apps or wearables collecting data about weight and physical activity [40,43]. According to a systematic content analysis, every third app did not provide a privacy policy [41]. Although app stores require a description covering several topics like imprint, responsibilities, and data privacy statement, there is no autonomous agency verifying the supply at its market. Therefore, the need of a standardized quality seal or system is growing, both to ensure comparability between apps and to provide security and reliability for users [40]. 

Besides the aforementioned deficiencies regarding data, there are also limitations with respect to expert involvement. A systematic review on medical apps for mobile phones revealed that there is a lack of expert involvement combined with a deficient adherence to medical evidence [44]. This also applies for apps used for overweight and obesity treatment. A study on the current weight management app market showed that apps lack in professional content expertise as well as in evidence-based online approaches [45]. In accordance, a scoping review on commercial mobile apps for weight management investigated the scientific quality of apps. It revealed that only 1% of nearly 400 included apps provided scientific evaluation and less than 0.5% indicated a health care expert involvement [46]. Moreover, a content analysis of more than 50 commercially available paediatric weight loss apps revealed that more than 60% of the evaluated apps lack expert recommendations [16]. This affects not only the app content itself, but also data privacy, as developers should ensure sufficient data protection. Data protection and data integrity require sufficient technical and data protection law expertise [47]. This leads to the demand for a high-quality, evidence-based app development process considered as a collaborative process between developers, researchers, clinicians, and users [46]. Moreover, the involvement of experts at an early stage of the development process is recommended and required [44]. Aforementioned concerns apply not only to apps, but also to wearables, especially with regard to data management (reliability, safety, and security) [48]. 

## 4. Digital Self-Monitoring of Diet and Physical Activity 

Diet tracking can be performed through apps on smartphones and watches, containing nutritional assessment methods like diet records, recalls, and food frequency questionnaires [49]. Furthermore, apps represent a time- and cost-effective method for the collection of health-related data with the potential of a broad dissemination and scalability [50,51,52]. In Germany, around every third person has a health app installed on the smartphone [53]. According to a population-based survey among more than 4.000 German adults, more than 60% of participants use smartphones. Of those, 20% use health apps, primarily focusing on smoking cessation, healthy diet, and weight loss [54]. Besides the use of apps for dietary self-monitoring and -management, there is also a recent proliferation of the “quantified self” movement with regard to wearable technologies. Ancillary devices like smart watches and fitness trackers can be connected to, e.g., smartphones for monitoring life attributes like diet, physical activity, and sleep and finally providing real-time feedback to the user [9]. Nowadays, wearable devices enable the tracking of numerous variables, e.g., blood pressure, blood glucose, and heart rate [9] by the application of novel technologies, like the non-invasive glucose monitoring by smart watches [55], smart patches [56], and smart clothes [57]. 

**Figure 2:**
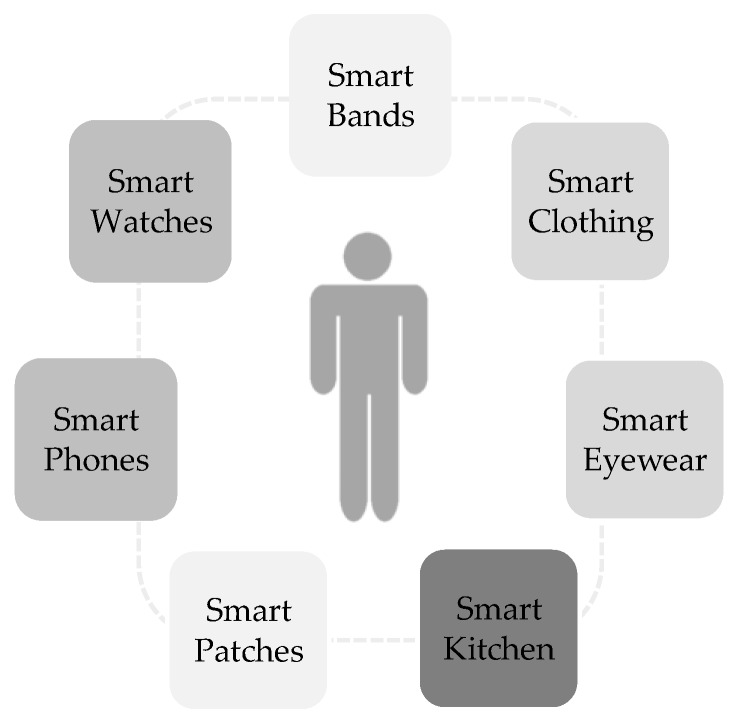
Examples of smart digital tools for monitoring of lifestyle and physiology.

Figure 2 provides an overview of selected smart technologies currently used for lifestyle monitoring. For instance, physical activity is mainly monitored by either wearable sensors (accelerometers) as wrist-worn bands, or apps for smartwatches or smartphones assessing GPS (global positioning system) data [58]. In the following, the focus is on apps and wearable technologies (activity trackers) for the purpose of weight management. 

## 5. Smartphone Applications (Apps) and Wearable Devices (Activity Tracker) for Weight Management

Systematic reviews and meta-analyses have investigated and proven the effectiveness of mobile phone and app-based interventions on weight-related outcomes [59,60,61,62,63,64,65]. Current evidence for the long-term effectivity of apps and activity trackers on weight management is limited due to inconsistent findings and a low methodological quality within studies [66]. A selection of studies, which investigated the long-term effects (>6 months) of apps and wearables on weight change are addressed in the following. 

Some studies compare in-person contact (e.g., phone calls, group sessions) against technology (app), combine both, or examine different modes of dietary self-monitoring with regard to weight change. One long-term randomized controlled trial (RCT) is the “Cell Phone Intervention for You (CITY)” study in which Svetkey et al. (2015) investigated two behavioral weight loss interventions among 365 young adults with overweight and obesity. The “cell phone” (CP) group was provided with a smartphone app which included a variety of behavioral change techniques, e.g., self-monitoring (diet, physical activity, and weight) and in-app prompting. The “personal coaching” (PC) group attended group sessions and received phone calls from coaches in addition to app-assisted self-tracking of weight, diet, and physical activity, but no in-app prompting [51,67]. Results revealed that CP participants had no weight loss advantages compared to controls at any time point (6, 12, and 24 months), while weight loss after 6 months was significantly higher (1.92 kg; CI 3.17, 0.67; *p* = 0.003) in the PC group than in the control group. Differences in mean weight loss at 24 months were not significant between intervention groups [51]. Besides that, a further publication about the CITY study from 2018 revealed that there was an association between engagement and weight loss during the first 6 intervention months [67]. Compared to PC participants, engagement and self-monitoring of weight were higher in CP participants. In general, engagement was the highest within the first months with a decreasing trend until the study end [67]. Besides the aforementioned interventions combining both digital and personal weight loss approaches, there are also studies focusing on each as a single approach. An 18 month RCT among 276 adults with overweight and obesity compared two behavioral weight loss approaches (group sessions, smartphone app) with controls [68]. While the more intense group-based intervention consisted of regular meetings declining in frequency over the study period, the app-based intervention included online lessons. Both groups performed self-monitoring of diet and received feedback, either paper-based or digital. Estimated mean weight change was similar in the intervention (5.9 kg; 95% CI 4.5, 7.4 vs. 5.5 kg; 95% CI 3.9, 7.1) and the control group (6.4 kg; 95% CI 3.7, 9.2) after 18 months. Thus, authors concluded that an app-based behavioral weight loss treatment can be as effective as a face-to-face group-based approach [68].

Two different mobile dietary self-monitoring tools (smartphone app, bite counter device) for weight loss were compared within the Dietary Intervention to Enhance Tracking with Mobile Devices (DIET Mobile) study among 81 overweight adults [69]. Besides the different tracking tools, both groups were provided with the same behavioral weight loss information delivered by podcasts. After 6 months, both intervention groups showed weight loss, with the app group participants loosing significantly more weight (−6.8  ±  0.8 kg; *p* < 0.001) than participants wearing the bite counter device (−3.0  ±  0.8 kg). There were no between-group differences regarding the frequency of dietary self-monitoring. Moreover, total weight loss was significantly correlated with frequency of information acquisition (podcasts) and self-monitoring (diet) [69]. Adherence and engagement to self-monitoring often appears in the early stage of interventions and declines continuously over time [67,70,71]. Different types of self-monitoring were evaluated by another intervention study among 128 participants with overweight. It was shown that weight loss was associated with the type of self-monitoring of diet (app, paper diary, and website) [52]. After six months, there was weight loss in all three groups, with the app being most effective (−4.6 kg; 95% CI −6.2, −3.0), followed by paper diary (−2.9 kg; 95% CI −4.7, −1.1) and the website group (−1.3 kg, 95% CI −2.7, 0.1). Study retention and self-monitoring adherence was highest for participants recording diet by using the smartphone app [52]. Furthermore, it has been shown that the daily frequency of dietary self-monitoring (log-in data website) is related to weight loss. However, daily duration of dietary self-monitoring is not associated with weight loss [6]. Outcomes need to be interpreted with caution, as there was no control group considered and results are based on log-in data only.

A recent meta-analysis revealed that, compared with control conditions, physical activity apps can increase daily steps, with a mean non-significant between-group difference of 476.75 steps per day (95% CI −229.57, 1183.07; *p* = 0.19) [72]. There is also evidence regarding the positive effects of wearables on physical activity and weight. A meta-analysis of nine RCTs and prospective cohort studies without dietary interventions showed that interventions with a pedometer increase steps and promote weight loss as well [73]. Compared to baseline, the mean weight change was −1.27 kg (CI 95%; −1.85, −0.70 kg). The average weight loss per week was 0.05 kg, with greater weight loss at longer intervention periods [73]. Evidence for the effectiveness of pedometers could also be demonstrated by a former systematic review, revealing that their application is associated with significant improvements of physical activity, body mass index (BMI), and blood pressure [74]. According to the included RCTs, it was shown that pedometer usage significantly increased daily physical activity by nearly 2500 steps more than under the control condition (95% CI; 1.098–3.885 steps/day; *p* < 0.001). This also applied for observational studies. In total and compared to baseline, the application of pedometers resulted in a more than 25% increase in physical activity. Moreover, BMI was significantly improved by a mean change of 0.38 (95% CI; 0.05, 0.72; *p* = 0.03) among pedometer users from pre- to post intervention. As the mean intervention duration was rather short (18 weeks), no long-term conclusions can be drawn [74]. Another systematic review by Lewis et al. showed preliminary evidence for wearables (activity tracker) and demonstrated that their application can lead to significant improvements of physical activity and weight from pre- to post intervention. Quality assessment revealed that most studies were of medium quality, concluding that there is need for more high-quality RCTs [75]. Furthermore, a systematic review by Goode et al. showed that wearable motion sensing technologies (accelerometers) had significant effects on improvements of both physical activity and weight loss. As effects were small and studies had only small sample sizes with moderate to high heterogeneity, results have to be interpreted cautiously with respect to clinical relevance [76]. 

Finkelstein et al. investigated the effectiveness of activity trackers combined with incentives among a subset of 800 employees in four intervention groups, activity tracker as a stand-alone intervention, activity tracker combined with cash incentive, activity tracker combined with charity incentive, and control [77]. Compared to controls, increase in Moderate-to-Vigorous Physical Activity (MVPA) was significantly greater in the tracker groups combined with cash or charity after the 6 month intervention. There was no significant difference in MVPA increase between the stand-alone activity tracker group and the control group. The 12 month post intervention follow-up revealed that compared with no treatment, all intervention modes with tracker increased MVPA. Finally, no improvements on health (weight, blood pressure, and quality of life measures) were detected at 6 or 12 months [77]. The Innovative Approaches to Diet, Exercise and Activity (IDEA) study compared long-term effects of a standard behavioral against a wearable-enhanced (commercially available) lifestyle intervention on weight loss among 471 adults with overweight and obesity [78]. Prior to randomization, participants were instructed to reduce caloric intake, to increase physical activity and received group counselling sessions for 6 months. At 6 months, further intervention elements (telephone counselling sessions, text message prompts, web-based study materials) were added. Hereafter, randomization to either the standard (web-based) or the wearable enhanced intervention group (wearable plus web interface) was performed in order to self-monitor diet and physical activity for another 18 months. After 24 months and compared to baseline, there was a significant weight change difference between both intervention groups by 2.4 kg (95% CI 1.0, 3.7; *p* = 0.002), with higher weight loss in the standard intervention group [78]. 

## 6. Summary and Outlook

Digital health applications and devices have become increasingly popular among the population. At the same time, the market for apps and wearables is growing rapidly with almost no external (e.g., federal) regulations. Several reports investigated the current market supply and found out that the main concerns relate to data safety, privacy, and transparency. In addition, the development of apps and wearables is often non-evidence based and without any expert involvement. Although there are several approaches for a harmonized evaluation of apps, there are no standardized criteria available by now. Besides that, studies evaluating the efficacy of digital tools regarding health-related outcomes have proliferated. According to the studies discussed in this article, interventions with apps and wearables are mainly not superior to non-digital treatments with regard to weight loss. Nevertheless, some studies addressed herein showed that the use of apps and wearables for self-monitoring of diet and physical activity promotes weight loss among adults, especially in combination with personal contact.

It has to be mentioned that weight outcomes within digitally (enhanced) interventions should be interpreted cautiously as weight is often assessed by self-report. This also applies for the assessment of diet and physical activity, which is usually self-reported, too. In accordance with non-digital interventions, digitally (enhanced) interventions are also affected by high attrition rates. Therefore, it is often impossible to evaluate their real impact as participants` compliance often decreases within the study progress [43]. Therefore, several approaches for the enhancement of self-monitoring adherence were examined [67,77,79,80]. For instance, Finkelstein et al. demonstrated that adherence to activity tracking benefits from the supportive application of incentives [77]. Besides that, there is evidence for the efficacy of technology-based reminders or prompts for engagement with digital intervention, which should be considered as well [79]. 

Most guidelines for the treatment of overweight and obesity do not refer to digital technology (internet, telephone, apps) as potential supportive treatment approaches. This might be due to the fact that the results of most RCTs on digital devices for weight loss have been published after the publication of the guidelines. The German “Interdisciplinary Guideline of Quality S3 for the Prevention and Treatment of Obesity” carefully considers apps as a synergistic and supportive tool for on-site programs [81]. Although digital tools have several benefits (e.g., availability, flexibility, and cost and time-effectiveness) compared to conventional face-to-face interventions, the German guideline claims that professional weight reduction programs without digital involvement are more successful with respect to therapeutic outcomes. As physicians claim that time pressure and costs are significant barriers within consultations [82], a digitally (supported) intervention might reduce the workload in the everyday practice life. According to the described studies, there is no clinical evidence to recommend apps as a standalone program for long-term weight loss purposes. As present work is intended to provide insights from different perspectives into the scientific research of apps and wearables for weight management, no systematic literature search or meta-analysis was conducted.

Finally, it is indispensable to conduct large intervention studies in order to generate valid recommendations for the use of apps and electronic devices for weight management. More research is needed to evaluate digital approaches of delivering lifestyle interventions for weight loss and weight maintenance. Aforementioned topics are addressed within the *enable* Nutrition Cluster (www.enable-cluster.de), funded by the German Federal Ministry of Education and Research. The aim of the corresponding lifestyle intervention (LION) study is to compare two dietary recommendations (low carbohydrate diet, low fat diet) and two different tools (app, newsletter) for weight loss maintenance. 

## References

[1] Abarca-Gómez L., Abdeen Z.A., Hamid Z.A., Abu-Rmeileh N.M., Acosta-Cazares B., Acuin C., Adams R.J., Aekplakorn W., Afsana K., Aguilar-Salinas C.A. (2017). Worldwide trends in body-mass index, underweight, overweight, and obesity from 1975 to 2016: A pooled analysis of 2416 population-based measurement studies in 128·9 million children, adolescents, and adults. Lancet.

[2] Wyatt S.B., Winters K.P., Dubbert P.M. (2006). Overweight and obesity: Prevalence, consequences, and causes of a growing public health problem. Am. J. Med. Sci..

[3] World Health Organization (WHO) Obesity and Overweight. https://www.who.int/news-room/fact-sheets/detail/obesity-and-overweight.

[4] Kohl H.W., Craig C.L., Lambert E.V., Inoue S., Alkandari J.R., Leetongin G., Kahlmeier S. (2012). The pandemic of physical inactivity: Global action for public health. Lancet.

[5] Stevens G.A., Singh G.M., Lu Y., Danaei G., Lin J.K., Finucane M.M., Bahalim A.N., McIntire R.K., Gutierrez H.R., Cowan M. (2012). National, regional, and global trends in adult overweight and obesity prevalences. Popul. Health Metr..

[6] Harvey J., Krukowski R., Priest J., West D. (2019). Log Often, Lose More: Electronic Dietary Self-Monitoring for Weight Loss. Obesity (Silver Spring).

[7] Carroll J.K., Moorhead A., Bond R., LeBlanc W.G., Petrella R.J., Fiscella K. (2017). Who Uses Mobile Phone Health Apps and Does Use Matter? A Secondary Data Analytics Approach. J. Med. Internet Res..

[8] Adjekum A., Blasimme A., Vayena E. (2018). Elements of Trust in Digital Health Systems: Scoping Review. J. Med. Internet Res..

[9] Davis T.L., DiClemente R., Prietula M. (2016). Taking mHealth Forward: Examining the Core Characteristics. JMIR Mhealth Uhealth.

[10] World Health Organization mHealth: New Horizons for Health through Mobile Technologies. Global Observatory for eHealth Series—Volume 3.

[11] Oh H., Rizo C., Enkin M., Jadad A. (2005). What is eHealth (3): A systematic review of published definitions. J. Med. Internet Res..

[12] U.S. Food and Drug Administration Digital Health. https://www.fda.gov/medicaldevices/digitalhealth/.

[13] Braz V.N., Lopes M.H.B.M. (2018). Evaluation of mobile applications related to nutrition. Public Health Nutr..

[14] Albrecht U.-V., Engeli S., von Jan U. (2016). Mit Apps durch Dick und Dünn?. Adipositas Ursachen Folgeerkrankungen Therapie.

[15] Pagoto S., Bennett G.G. (2013). How behavioral science can advance digital health. Transl. Behav. Med..

[16] Schoffman D.E., Turner-McGrievy G., Jones S.J., Wilcox S. (2013). Mobile apps for pediatric obesity prevention and treatment, healthy eating, and physical activity promotion: Just fun and games?. Transl. Behav. Med..

[17] Chen J., Cade J.E., Allman-Farinelli M. (2015). The Most Popular Smartphone Apps for Weight Loss: A Quality Assessment. JMIR Mhealth Uhealth.

[18] Bardus M., van Beurden S.B., Smith J.R., Abraham C. (2016). A review and content analysis of engagement, functionality, aesthetics, information quality, and change techniques in the most popular commercial apps for weight management. Int. J. Behav. Nutr. Phys. Act..

[19] Becker S., Miron-Shatz T., Schumacher N., Krocza J., Diamantidis C., Albrecht U.-V. (2014). mHealth 2.0: Experiences, Possibilities, and Perspectives. JMIR Mhealth Uhealth.

[20] Holzmann S.L., Pröll K., Hauner H., Holzapfel C. (2018). Nutrition apps: Quality and limitations: An explorative investigation on the basis of selected apps. Ernahrungs Umschau.

[21] Federal Institute for Drugs and Medical Devices BfArM—Guidance on “Medical Apps”: Differentiation between Apps and Medical or Other Devices as well as on the Subsequent Risk Classification According to the MPG. https://www.bfarm.de/EN/MedicalDevices/Differentiation/MedicalApps/_node.html.

[22] European Commission (2014). Green Paper on Mobile Health (“mHealth”). Ec.europa.eu/newsroom/dae/document.cfm?doc_id=5147.

[23] European Commission Privacy Code of Conduct on Mobile Health Apps. https://ec.europa.eu/digital-single-market/en/privacy-code-conduct-mobile-health-apps.

[24] Agarwal S., LeFevre A.E., Lee J., L’Engle K., Mehl G., Sinha C., Labrique A. (2016). Guidelines for reporting of health interventions using mobile phones: Mobile health (mHealth) evidence reporting and assessment (mERA) checklist. BMJ.

[25] Xcertia Xcertia-mHealth App Guidelines. https://www.xcertia.org/the-guidelines/.

[26] Stoyanov S.R., Hides L., Kavanagh D.J., Zelenko O., Tjondronegoro D., Mani M. (2015). Mobile app rating scale: A new tool for assessing the quality of health mobile apps. JMIR Mhealth Uhealth.

[27] DiFilippo K.N., Huang W., Chapman-Novakofski K.M. (2017). A New Tool for Nutrition App Quality Evaluation (AQEL): Development, Validation, and Reliability Testing. JMIR Mhealth Uhealth.

[28] Albrecht U.-V. (2013). Transparency of health-apps for trust and decision making. J. Med. Internet Res..

[29] Albrecht U.-V., Hillebrand U., von Jan U. (2018). Relevance of Trust Marks and CE Labels in German-Language Store Descriptions of Health Apps: Analysis. JMIR Mhealth Uhealth.

[30] Kramer U. HealthOn-App Ehrenkodex für Gesundheits-Apps. https://www.healthon.de/ehrenkodex.

[31] Düsseldorfer Kreis Orientierungshilfe zu den Datenschutzanforderungen an App-Entwickler und App-Anbieter. https://www.baden-wuerttemberg.datenschutz.de/wp-content/uploads/2013/02/OH_Apps_20140616.pdf.

[32] Zentrum für Telematik und Telemedizin GmbH AppCheck: Die Informations- und Bewertungsplattform für Gesundheits-Apps. https://appcheck.de/technische-pruefung/.

[33] Fraunhofer-Institut für Offene Kommunikationssysteme APPKRI Kriterien für Gesundheits-Apps. https://ehealth-services.fokus.fraunhofer.de/BMG-APPS/impressum.

[34] Albrecht U.-V. Chances and Risks of Mobile Health Apps (CHARISMHA). https://charismha.weebly.com/uploads/7/4/0/7/7407163/charismha_abr_v.01.1e-20160606.pdf.

[35] Gigerenzer G., Schlegel-Matthies K., Wagner G.G. (2016). Digitale Welt und Gesundheit. eHealth und mHealth—Chancen und Risiken der Digitalisierung im Gesundheitsbereich. http://www.svr-verbraucherfragen.de/dokumente/digitale-welt-und-gesundheit-ehealth-und-mhealth-chancen-und-risiken-der-digitalisierung-im-gesundheitsbereich/.

[36] Aktionsbündnis Patientensicherheit e.V. (2018). Digitalisierung und Patientensicherheit. Checkliste für die Nutzung von Gesundheits-Apps. www.aps-ev.de/wp-content/uploads/2018/05/2018_APS-Checkliste_GesundheitsApps.pdf.

[37] Knöppler K., Neisecke T., Nölke L. Digital-Health-Anwendungen für Bürger. Kontext, Typologie und Relevanz aus Public-Health-Perspektive Entwicklung und Erprobung eines Klassifikationsverfahrens. https://www.bertelsmann-stiftung.de/fileadmin/files/BSt/Publikationen/GrauePublikationen/Studie_VV_Digital-Health-Anwendungen_2016.pdf.

[38] Kramer U. (2017). Wie gut sind Gesundheits-Apps?. Aktuel Ernahrungsmed.

[39] Weichert T. (2018). Gesundheitsdatenschutz in vernetzten Zeiten. Bundesgesundheitsblatt Gesundheitsforschung Gesundheitsschutz.

[40] Hutton L., Price B.A., Kelly R., McCormick C., Bandara A.K., Hatzakis T., Meadows M., Nuseibeh B. (2018). Assessing the Privacy of mHealth Apps for Self-Tracking: Heuristic Evaluation Approach. JMIR Mhealth Uhealth.

[41] Das G., Cheung C., Nebeker C., Bietz M., Bloss C. (2018). Privacy Policies for Apps Targeted Toward Youth: Descriptive Analysis of Readability. JMIR Mhealth Uhealth.

[42] Sunyaev A., Dehling T., Taylor P.L., Mandl K.D. (2015). Availability and quality of mobile health app privacy policies. J. Am. Med. Inform. Assoc..

[43] Ellis D.A., Piwek L. (2018). Failing to encourage physical activity with wearable technology: What next?. J. R. Soc. Med..

[44] Subhi Y., Bube S.H., Rolskov Bojsen S., Skou Thomsen A.S., Konge L. (2015). Expert Involvement and Adherence to Medical Evidence in Medical Mobile Phone Apps: A Systematic Review. JMIR Mhealth Uhealth.

[45] Nikolaou C.K., Lean M.E.J. (2017). Mobile applications for obesity and weight management: Current market characteristics. Int. J. Obes..

[46] Rivera J., McPherson A., Hamilton J., Birken C., Coons M., Iyer S., Agarwal A., Lalloo C., Stinson J. (2016). Mobile Apps for Weight Management: A Scoping Review. JMIR Mhealth Uhealth.

[47] Albrecht U.-V., von Jan U. (2017). Safe, sound and desirable: Development of mHealth apps under the stress of rapid life cycles. Mhealth.

[48] Piwek L., Ellis D.A., Andrews S., Joinson A. (2016). The Rise of Consumer Health Wearables: Promises and Barriers. PLoS Med..

[49] McClung H.L., Ptomey L.T., Shook R.P., Aggarwal A., Gorczyca A.M., Sazonov E.S., Becofsky K., Weiss R., Das S.K. (2018). Dietary Intake and Physical Activity Assessment: Current Tools, Techniques, and Technologies for Use in Adult Populations. Am. J. Prev. Med..

[50] Roess A. (2017). The Promise, Growth, and Reality of Mobile Health—Another Data-free Zone. N. Engl. J. Med..

[51] Svetkey L.P., Batch B.C., Lin P.-H., Intille S.S., Corsino L., Tyson C.C., Bosworth H.B., Grambow S.C., Voils C., Loria C. (2015). Cell phone intervention for you (CITY): A randomized, controlled trial of behavioral weight loss intervention for young adults using mobile technology. Obesity (Silver Spring).

[52] Carter M.C., Burley V.J., Nykjaer C., Cade J.E. (2013). Adherence to a smartphone application for weight loss compared to website and paper diary: Pilot randomized controlled trial. J. Med. Internet Res..

[53] Thranberend T., Knöppler K., Neisecke T. (2016). Gesundheits-Apps. Bedeutender Hebel für Patient Empowerment—Potenziale Jedoch Bislang Kaum Genutzt. https://www.bertelsmann-stiftung.de/fileadmin/files/BSt/Publikationen/GrauePublikationen/SpotGes_Gesundheits-Apps_dt_final_web.pdf.

[54] Ernsting C., Dombrowski S.U., Oedekoven M., O Sullivan J.L., Kanzler M., Kuhlmey A., Gellert P. (2017). Using Smartphones and Health Apps to Change and Manage Health Behaviors: A Population-Based Survey. J. Med. Internet Res..

[55] Campbell M. Apple Patent Hints at Non-Invasive Glucose Monitoring Tech for Apple Watch. https://appleinsider.com/articles/18/08/23/apple-patent-suggests-work-on-non-invasive-glucose-monitoring-tech.

[56] Khan S., Ali S., Bermak A. (2019). Recent Developments in Printing Flexible and Wearable Sensing Electronics for Healthcare Applications. Sensors.

[57] Fernández-Caramés T., Fraga-Lamas P. (2018). Towards the Internet-of-Smart-Clothing: A Review on IoT Wearables and Garments for Creating Intelligent Connected E-Textiles. Electronics.

[58] Gilmore L.A., Duhé A.F., Frost E.A., Redman L.M. (2014). The technology boom: A new era in obesity management. J. Diabetes Sci. Technol..

[59] DiFilippo K.N., Huang W.-H., Andrade J.E., Chapman-Novakofski K.M. (2015). The use of mobile apps to improve nutrition outcomes: A systematic literature review. J. Telemed. Telecare.

[60] Aguilar-Martínez A., Solé-Sedeño J.M., Mancebo-Moreno G., Medina F.X., Carreras-Collado R., Saigí-Rubió F. (2014). Use of mobile phones as a tool for weight loss: A systematic review. J. Telemed. Telecare.

[61] Flores Mateo G., Granado-Font E., Ferré-Grau C., Montaña-Carreras X. (2015). Mobile Phone Apps to Promote Weight Loss and Increase Physical Activity: A Systematic Review and Meta-Analysis. J. Med. Internet Res..

[62] Liu F., Kong X., Cao J., Chen S., Li C., Huang J., Gu D., Kelly T.N. (2015). Mobile phone intervention and weight loss among overweight and obese adults: A meta-analysis of randomized controlled trials. Am. J. Epidemiol..

[63] Stephens J., Allen J. (2013). Mobile phone interventions to increase physical activity and reduce weight: A systematic review. J. Cardiovasc. Nurs..

[64] Schippers M., Adam P.C.G., Smolenski D.J., Wong H.T.H., de Wit J.B.F. (2017). A meta-analysis of overall effects of weight loss interventions delivered via mobile phones and effect size differences according to delivery mode, personal contact, and intervention intensity and duration. Obes. Rev..

[65] Park S.-H., Hwang J., Choi Y.-K. (2019). Effect of Mobile Health on Obese Adults: A Systematic Review and Meta-Analysis. Healthc. Inform. Res..

[66] Marcolino M.S., Oliveira J.A.Q., D’gostino M., Ribeiro A.L., Alkmim M.B.M., Novillo-Ortiz D. (2018). The Impact of mHealth Interventions: Systematic Review of Systematic Reviews. JMIR Mhealth Uhealth.

[67] Lin P.-H., Grambow S., Intille S., Gallis J.A., Lazenka T., Bosworth H., Voils C.L., Bennett G.G., Batch B., Allen J. (2018). The Association Between Engagement and Weight Loss Through Personal Coaching and Cell Phone Interventions in Young Adults: Randomized Controlled Trial. JMIR Mhealth Uhealth.

[68] Thomas J.G., Bond D.S., Raynor H.A., Papandonatos G.D., Wing R.R. (2019). Comparison of Smartphone-Based Behavioral Obesity Treatment with Gold Standard Group Treatment and Control: A Randomized Trial. Obesity (Silver Spring).

[69] Turner-McGrievy G.M., Wilcox S., Boutté A., Hutto B.E., Singletary C., Muth E.R., Hoover A.W. (2017). The Dietary Intervention to Enhance Tracking with Mobile Devices (DIET Mobile) Study: A 6-Month Randomized Weight Loss Trial. Obesity (Silver Spring).

[70] Burke L.E., Styn M.A., Sereika S.M., Conroy M.B., Ye L., Glanz K., Sevick M.A., Ewing L.J. (2012). Using mHealth technology to enhance self-monitoring for weight loss: A randomized trial. Am. J. Prev. Med..

[71] Zheng Y., Burke L.E., Danford C.A., Ewing L.J., Terry M.A., Sereika S.M. (2016). Patterns of self-weighing behavior and weight change in a weight loss trial. Int. J. Obes..

[72] Romeo A., Edney S., Plotnikoff R., Curtis R., Ryan J., Sanders I., Crozier A., Maher C. (2019). Can Smartphone Apps Increase Physical Activity? Systematic Review and Meta-Analysis. J. Med. Internet Res..

[73] Richardson C.R., Newton T.L., Abraham J.J., Sen A., Jimbo M., Swartz A.M. (2008). A meta-analysis of pedometer-based walking interventions and weight loss. Ann. Fam. Med..

[74] Bravata D.M., Smith-Spangler C., Sundaram V., Gienger A.L., Lin N., Lewis R., Stave C.D., Olkin I., Sirard J.R. (2007). Using Pedometers to Increase Physical Activity and Improve Health: A Systematic Review. JAMA.

[75] Lewis Z.H., Lyons E.J., Jarvis J.M., Baillargeon J. (2015). Using an electronic activity monitor system as an intervention modality: A systematic review. BMC Public Health.

[76] Goode A.P., Hall K.S., Batch B.C., Huffman K.M., Hastings S.N., Allen K.D., Shaw R.J., Kanach F.A., McDuffie J.R., Kosinski A.S. (2017). The Impact of Interventions that Integrate Accelerometers on Physical Activity and Weight Loss: A Systematic Review. Ann. Behav. Med..

[77] Finkelstein E.A., Haaland B.A., Bilger M., Sahasranaman A., Sloan R.A., Nang E.E.K., Evenson K.R. (2016). Effectiveness of activity trackers with and without incentives to increase physical activity (TRIPPA): A randomised controlled trial. Lancet Diabetes Endocrinol..

[78] Jakicic J.M., Davis K.K., Rogers R.J., King W.C., Marcus M.D., Helsel D., Rickman A.D., Wahed A.S., Belle S.H. (2016). Effect of Wearable Technology Combined with a Lifestyle Intervention on Long-term Weight Loss: The IDEA Randomized Clinical Trial. JAMA.

[79] Alkhaldi G., Hamilton F.L., Lau R., Webster R., Michie S., Murray E. (2016). The Effectiveness of Prompts to Promote Engagement with Digital Interventions: A Systematic Review. J. Med. Internet Res..

[80] Shaw R., Levine E., Streicher M., Strawbridge E., Gierisch J., Pendergast J., Hale S., Reed S., McVay M., Simmons D. (2019). Log2Lose: Development and Lessons Learned from a Mobile Technology Weight Loss Intervention. JMIR Mhealth Uhealth.

[81] Deutsche Adipositas-Gesellschaft (DAG) e.V., Deutsche Diabetes Gesellschaft (DDG), Deutsche Gesellschaft für Ernährung (DGE) e.V., Deutsche Gesellschaft für Ernährungsmedizin (DGEM) e.V. Interdisziplinäre Leitlinie der Qualität S3 zur “Prävention und Therapie der Adipositas”. https://www.adipositas-gesellschaft.de/fileadmin/PDF/Leitlinien/050-001l_S3_Adipositas_Praevention_Therapie_2014-11.pdf.

[82] Mazza D., McCarthy E., Carey M., Turner L., Harris M. (2019). “90% of the time, it’s not just weight”: General practitioner and practice staff perspectives regarding the barriers and enablers to obesity guideline implementation. Obes. Res. Clin. Pract..

